# Rational Design of Antifungal Peptides Based on the γ-Core Motif of a *Neosartorya* (*Aspergillus*) *fischeri* Antifungal Protein to Improve Structural Integrity, Efficacy, and Spectrum

**DOI:** 10.1021/acsomega.3c09377

**Published:** 2024-01-31

**Authors:** Györgyi Váradi, Gábor Bende, Attila Borics, Kinga Dán, Gábor Rákhely, Gábor K. Tóth, László Galgóczy

**Affiliations:** † Department of Medical Chemistry, University of Szeged, Szeged 6720, Hungary; ‡ Department of Biotechnology, University of Szeged, Szeged 6726, Hungary; § Doctoral School of Biology, University of Szeged, Szeged 6720, Hungary; ∥ Institute of Biochemistry, HUN-REN Biological Research Centre, Szeged 6726, Hungary; ⊥ Institute of Biophysics, HUN-REN Biological Research Centre, Szeged 6726, Hungary; # MTA-SZTE Biomimetic Systems Research Group, University of Szeged, Szeged 6720, Hungary

## Abstract

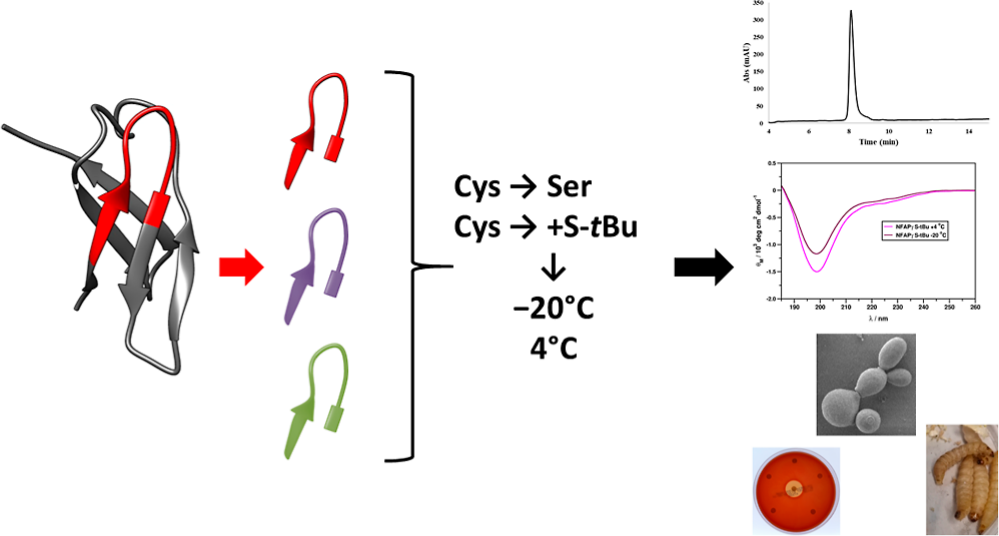

Antifungal peptides offer promising alternative compounds for the treatment of fungal infections, for which new antifungal compounds are urgently needed. Constant and broad antifungal spectra of these peptides play essential roles in their reliable therapeutic application. It has been observed that rationally designed peptides using the evolutionarily conserved γ-core region (GXC–X_3–9_–C) of an antifungal protein from *Neosartorya* (*Aspergillus*) *fischeri* highly inhibit the growth of fungi. The cysteines in these peptides have free sulfhydryl groups, which allow cyclization and dimerization under oxidative conditions, thereby impairing antifungal efficacy. To overcome this problem, one or two cysteine residues were substituted by serines or *S*-*tert*-butyl was applied as a cysteine-protecting group. Furthermore, structural integrity and antifungal efficacy investigations before and after oxidative exposure revealed that substituting both cysteines with serines and S-*tert*-butylation helped maintain the structural integrity. However, it slightly decreased the antifungal efficacy against a yeast, *Candida albicans*. Interestingly, S-*tert*-butylation maintained the efficacy and could extend the antifungal activity to a mold, *Aspergillus fumigatus*. Usually, cyclization and dimerization did not influence the antifungal efficacy of most peptides. Additionally, hemolysis tests and *Galleria mellonella* toxicity model experiments indicated that none of the applied modifications made the peptides harmful to animals.

## 1 Introduction

Currently, fungal infections are overlooked problems and have received less public attention.^1^ Nevertheless, recent epidemiological surveys have shed light on the increasing incidence and widespread of (multi)drug resistance to the four currently used antifungal agent classes, such as azoles, polyamines, pyrimidines, and echinocandins. Due to the lack of effective antifungals, the mortality rates of fungal infections remain high, especially among immunocompromised patients.^2^ Furthermore, prolonged therapeutic antifungal drug application is necessary, which is associated with severe side effects in most cases.^3,4^ Considering these alarming facts, the World Health Organization brought this problem to public attention and highlighted 19 fungal species (which pose the most severe risk to human health) on the fungal priority pathogen list (FPPL) of 2022. Besides, FPPL indicates required action areas, including enhanced public health interventions to prevent and control infection and the emergence of antifungal drug resistance.^5,6^ The main requirements for new antifungal drug development must be less toxic to the host and have a broad spectrum, fungal-targeted mode of action, and different mode of action from the existing agents to limit the potential for the emergence of resistance. Although the features of several novel compounds meet these requirements, only a few have reached the clinical trial phases.^7,8^ Therefore, the development of new antifungal therapeutic molecules is still in urgent demand to overcome the recent antifungal drug crisis.^9^

The features of natural, synthetic, and semisynthetic antifungal peptides (AFPs) make them promising powerful alternatives and bases of new antifungal strategies.^10,11^ AFPs exert their activities through different modes of action, and most of them have multiple antifungal mechanisms based on the applied concentrations. The simplest one is their rapid interaction with fungal cell membranes at high concentrations, causing cell leakage and death. More complex mechanisms are mainly observed at lower concentrations, such as cell wall synthesis inhibition, nucleic acid binding, mitochondrial dysfunction, production of reactive oxygen species, programmed cell death, autophagy, vacuolar dysfunction, cation homeostasis disruption, adenosine triphosphate efflux, and cell cycle impairment.^12^ Rapid membrane disruption and more complex modes of action can prevent a fast resistance development. Other advantages are the high fungal selectivity, low host toxicity, and broad antifungal spectrum.^11^ Despite these advantages, only a few AFPs have been brought to clinical trials (*e.g.*, nikkomycin Z, aureobasidin A, and VL-239) because of some unfavorable characteristics, limiting their application as antifungal drugs.^10^ These include instability due to host enzyme degradation, poor pharmacodynamic and pharmacokinetic properties compared with conventional drugs, unsolved routes of administration, low yield production, and high production cost.^12,13^ The recent new solid- and solution-phase peptide synthesis techniques can reduce the production cost and overcome the challenges of antimicrobial peptide production by biological expression systems,^14^ such as low yield, degradation, and activity loss.^15^ Rational peptide design, chemical modification, and development of drug delivery systems can improve the stability and bioavailability of AFPs.^11^

In our previous studies, we demonstrated that a synthetic peptide (NFAPγ) with the evolutionarily conserved γ-core region of the *Neosartorya* (*Aspergillus*) *fischeri* antifungal protein (NFAP) did not exhibit antifungal activity. However, its rationally designed variants with elevated net charge (*i.e.*, NFAPimpγ and NFAPimpγGZ in Table 1) effectively inhibit the growth of several plant pathogenic filamentous fungi.^16,17^ Presumably, these peptides exert an antifungal mechanism through membrane disruption,^17^ and the high positive net charge supports this mechanism. Additionally, neither hydrophilicity nor primary structure influences the antifungal activity.^16^ Hence, fungal-specific membrane targets may be responsible for the membrane disruption effect of NFAPimpγ and NFAPimpγGZ. This assumption is supported by the fact that these compounds did not exhibit significant toxic effects on mammalian cell lines and did not cause hemolysis.^16^ Thus, NFAPimpγ and NFAPimpγGZ represent promising antifungal compounds. However, several freeze–thaw cycles influence structural integrity, impairing their antifungal efficacy. Both peptides possess two cysteine residues with free sulfhydryl groups, which can form disulfide bridges under oxidative conditions, causing multimerization and/or cyclization. It was previously observed that such cyclization can impair the antifungal efficacy of a *Penicillium chrysogenum* AFP γ-core peptide derivative.^18^ For the reliable long-term application of antifungal active AFPs (*e.g.*, NFAPimpγ and NFAPimpγGZ), it is necessary to ensure their structural integrity and constant antifungal efficacy. Cysteine–serine substitutions and application of the *S*-*tert*-butyl (*S*-*t*Bu) protecting group on cysteine residues can avoid disulfide bridge formation, thereby facilitating structural integrity. However, it is unknown how these modifications influence antifungal efficacy. In this study, we synthesized various cysteine–serine substituted and *tert*-butylated NFAP γ-core peptide derivatives (Table 1) to address this issue. Furthermore, we investigated how these modifications maintain their structural integrity, influence fungal specificity, and alter antifungal efficacy.

**Table 1:** Physicochemical Properties of *Neosartorya* (*Aspergillus*) *fischeri* Antifungal Protein (NFAP) γ-Core Peptide Derivatives

peptide	number of amino acids	*M*_w_ (Da)	number of Cys	number of Lys/Arg/His	theoretical pI	charge at pH 7	GRAVY^a^
Ac-EYKGEC(–SH)FTKDNTC(–SH)K-NH_2_^b^							
NFAPγ^c^	14	1707.879	2	3/0/0	6.26	–0.1	–1.500
Ac-EYKGECFTKDNTSK-NH_2_^b^							
NFAPγ^C13S^	14	1691.814	1	3/0/0	6.27	–0.1	–1.736
Ac-EYKGESFTKDNTSK-NH_2_^b^							
NFAPγ^C6S,C13S^	14	1675.749	0	3/0/0	6.28	–0.1	–1.971
Ac-EYKGEC(-StBu)FTKDNTC(-StBu)K-NH_2_^b^							
NFAPγ^S-*t*Bu^	14	1820.108	2	3/0/0	n.d.	n.d.	n.d.
Ac-EYKGKC(–SH)KTKKNKC(–SH)K-NH_2_^b^							
NFAPimpγ^c^	14	1728.089	2	7/0/0	9.84	+5.8	–2.264
Ac-EYKGKCKTKKNKSK-NH_2_^b^							
NFAPimpγ^C13S^	14	1712.025	1	7/0/0	10.02	+5.9	–2.500
Ac-EYKGKSKTKKNKSK-NH_2_^b^							
NFAPimpγ^C6S,C13S^	14	1695.960	0	7/0/0	10.22	+5.9	–2.736
Ac-EYKGKC(-StBu)KTKKNKC(-StBu)K-NH_2_^b^							
NFAPimpγ^S-*t*Bu^	14	1840.318	2	7/0/0	n.d.	n.d.	n.d.
Ac-EIKIKC(–SH)KIKKIKC(–SH)K-NH_2_^b^							
NFAPimpγGZ^c^	14	1745.291	2	7/0/0	9.93	+5.8	–0.557
Ac-EIKIKCKIKKIKSK-NH_2_^b^							
NFAPimpγGZ^C13S^	14	1729.226	1	7/0/0	10.14	+5.9	–0.793
Ac-EIKIKSKIKKIKSK-NH_2_^b^							
NFAPimpγGZ^C6S,C13S^	14	1713.162	0	7/0/0	10.40	+5.9	–1.029
Ac-EIKIKC(-StBu)KIKKIKC(-StBu)K-NH_2_^b^							
NFAPimpγGZ^S-tBu^	14	1857.520	2	7/0/0	n.d.	n.d.	n.d.

a GRAVY: grand average of hydropathy value.

b Ac-: N-terminal acetylation, (–SH): free sulfhydryl group of cysteine, –NH_2_: C-terminal amidation.

c Described as γNFAP, γNFAP-opt, and γNFAP-optGZ, respectively, by Tóth *et al.* (2020).^16^ n.d.: data not available.

## 2 Results and Discussion

### 2.1 Peptide Design, Synthesis, and Physicochemical Properties

NFAPγ peptide was designed according to the native γ-core motif of NFAP (GECFTKDNTC), as previously described by Tóth *et al.*. (2020).^16^ It contains three additional amino acids (EYK) from the N-terminus and an extra lysine (K) from the C-terminus. To provide stability against proteolytic degradation and neutral termini, the N-terminus of this peptide is acetylated and the C-terminus is amidated (Table 1). These modifications provide propagation of the native protein backbone. NFAPγ is almost neutral (total net charge is −0.1 at pH = 7.0) and hydrophilic (grand average of hydropathy value, GRAVY: −1.500). Its rationally designed variant with an elevated net charge, NFAPimpγ (net charge is +5.8 at pH = 7.0, GRAVY: −2.264), and its increased GRAVY variant, NFAPimpγGZ (net charge is +5.8 at pH = 7.0, GRAVY: −0.557), were designed with the same considerations (Table 1). All cysteine residues in these three peptides possessed free sulfhydryl groups (Tóth *et al.*., 2020).^16^ Therefore, to avoid cyclization and multimerization by disulfide bridge formation between the sulfhydryl groups of cysteine residues, one of the cysteines or both were substituted with serine (C13S and C6S, C13S variants, respectively), or both were protected by S-*tert*-butylation (S-*t*Bu variants). None of these modifications changed the net charge. However, the cysteine–serine substitution decreased the GRAVY, whereas S-*tert*-butylation increased it, making the peptide more and less hydrophilic, respectively (Table 1).

**Figure 1:**
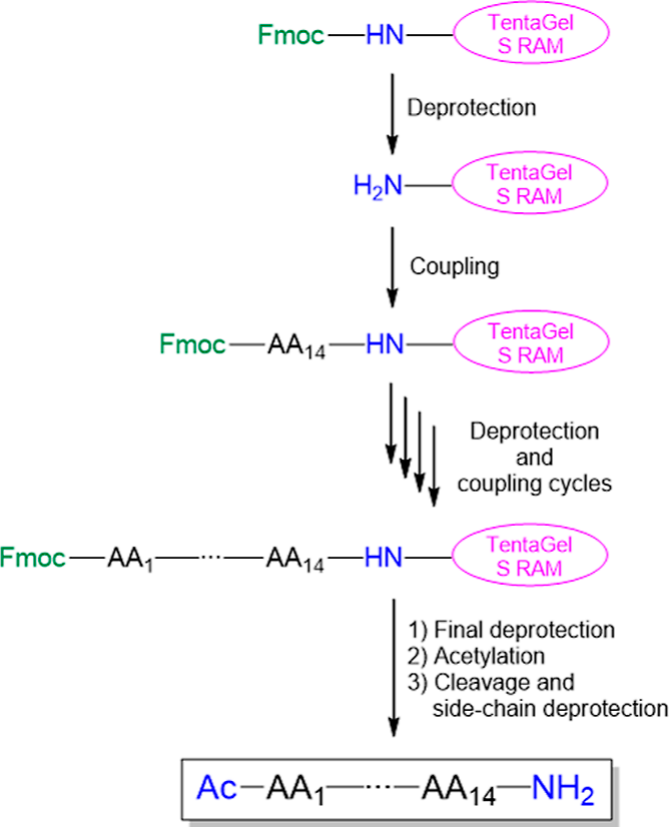
Solid-phase synthesis of NFAPγ peptides and their variants.

To prepare peptide amides, TentaGel S RAM resin was used as a solid support. The syntheses were performed using the microwave-assisted method following the standard 9-fluorenylmethoxycarbonyl (Fmoc)-based protocol. Before the cleavage of the peptides from the resin, the N-terminal free amino group was acetylated. These steps are summarized in Figure 1. The crude products were analyzed by using reversed-phase high-performance liquid chromatography (RP-HPLC) and mass spectrometry and purified.

### 2.2 Structural Integrity

The half volume of the dissolved lyophilized peptides was promptly stored at −20 °C for 7 days. The other half volume of these peptides was stored at 4 °C for 7 days (oxidative condition) and then at −20 °C until RP-HPLC and electronic circular dichroism (ECD) analyses were performed. Herein, these peptide samples are referred to as −20 and 4 °C samples, respectively.

RP-HPLC analysis showed that the NFAPγ peptide was highly prone to disulfide bridge formation. In 7 days at 4 °C, most of this peptide formed intramolecular, whereas a smaller part formed an intermolecular disulfide bond. Cyclic peptide and dimer formation can be observed, even in the −20 °C sample (Figure S1). The cysteine-serine-substituted and S-*t*Bu variants of NFAPγ remained unchanged under oxidative conditions (Figure S1). NFAPimpγ, the more basic and hydrophobic variant of the original γ-core peptide, had a stronger tendency to form disulfide bridges. The total amount of the peptide was cyclized at 4 °C for 7 days. Even at −20 °C, the sulfhydryl groups of the cysteines in approximately 60% of the peptide formed intramolecular disulfide bridges (Figure S2). NFAPimpγ^C13S^ had a slightly lower but still high propensity to be oxidized. In the 4 °C sample, approximately 60% of the peptide was in dimer form after 7 days (Figure S2). In the series of NFAPimpγGZ, the peptide possessed physicochemical properties similar to those of NFAPimpγ but close to zero hydrophobicity, and only the two cysteine-containing variants were oxidized (Figure S3). In 7 days at 4 °C, NFAPimpγGZ was fully oxidized to the cyclic form. At −20 °C, approximately one-third of the peptide formed intramolecular, and the other one-third formed intermolecular disulfide bridges (Figure S3). Based on the RP-HPLC investigation of the peptide samples, all three peptides containing two cysteines were almost completely or completely oxidized to the cyclic form by intramolecular disulfide bond formation at 4 °C for 7 days (Figures S1–S3).

**Figure 2:**
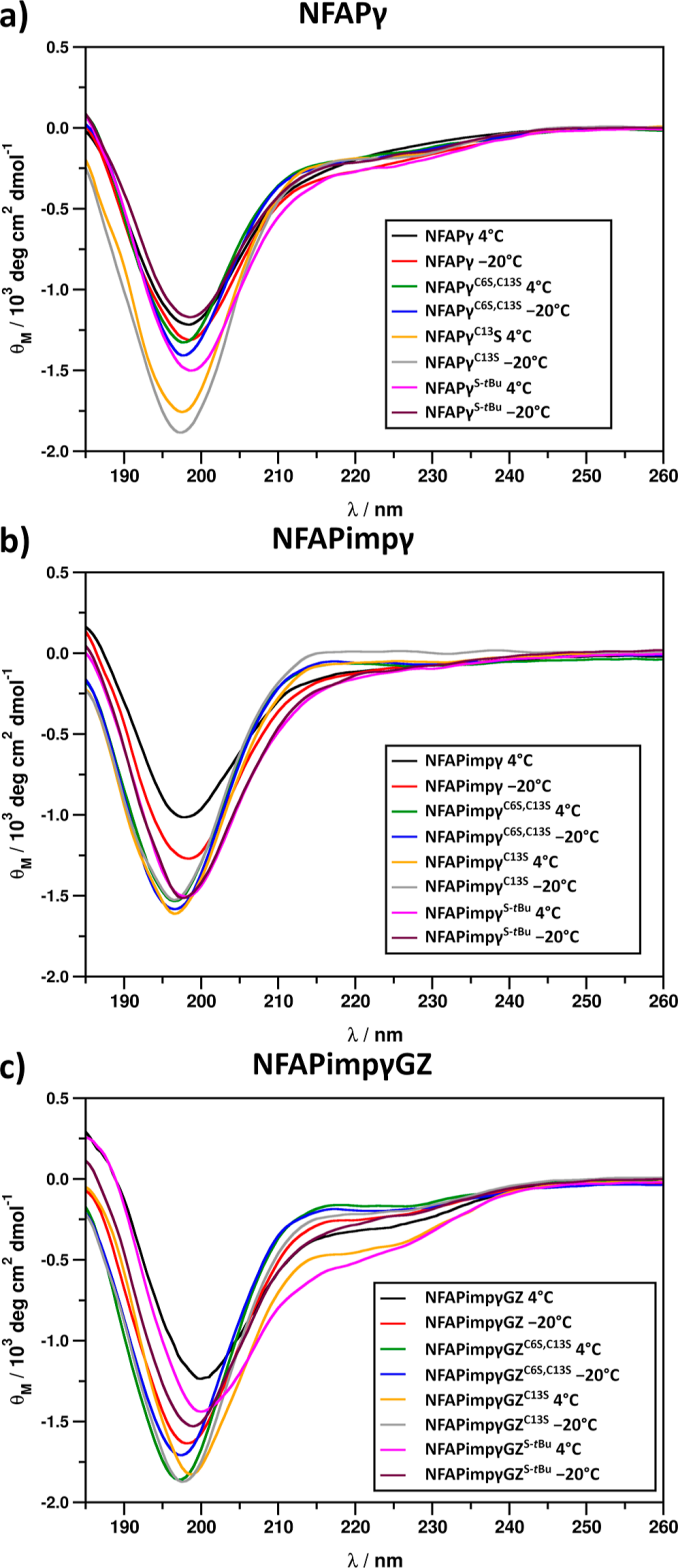
ECD spectra of −20 and 4 °C samples of various NFAP γ-core peptide derivatives (a–c).

ECD spectra of all NFAP γ-core peptide derivatives, including the parent peptide, exhibited unordered structures (Figure 2a–c) similar to those reported previously for peptide fragments and derivatives of similar origin.^18−20^ Moderate intensity differences were observed for spectra recorded for various samples. Peptides of this size usually adopt multiple structural states in solution, and the equilibrium of these states dictates the development of the corresponding ECD spectrum, which is the combination of contributions emerging from all of the present structural states. Therefore, the slight differences observed in spectral intensities (Figure 2a–c) could be attributed to variations in the solution conformational equilibria of the different peptides. The other reason for the spectral intensity differences (Figure 2a–c) could be the degradation or transformation of samples of various peptides stored under different conditions. Apart from intensity distinctness, all NFAPγ (Figure 2a) and NFAPimpγ (Figure 2b) spectra exhibited the same essential features of flexible and unordered structures. For the NFAPimpγGZ peptides (Figure 2c), minor contributions emerging from the helical structures were observed. Such contributions were most evident for the C13S and *S*-*t*Bu derivatives stored under oxidative conditions. However, such a substantial increase in helical contributions was not clearly supported by the secondary structure obtained from the circular dichroism spectra (CDSSTR) analysis of ECD (Table S1). This suggests that the structural differences that induced the observed spectral features are minor.

### 2.3 Antifungal Susceptibility

The peptide derivative spanning the native NFAP γ-core motif (NFAPγ), and its variants were inactive against the tested fungi, *Aspergillus fumigatus* and *Candida albicans* (minimum inhibitory concentration, MIC: >200 μg mL^–1^) (data not shown). The NFAPimpγ peptides inhibited the growth of *C. albicans* with MICs from 12.5 to 50 μg mL^–1^; however, they were inactive against *A. fumigatus*, except NFAPimpγ^S-*t*Bu^ (MIC: 25 μg mL^–1^). The NFAPimpγGZ peptides proved to be antifungal active against both fungi, with MICs from 12.5 to 50 μg mL^–1^. The seven-day-long storage at 4 °C did not influence the antifungal efficacy of the peptides; however, an increase in the MIC of NFAPimpγ from 12.5 to 25 μg mL^–1^ was observed. Substitutions of cysteines with serines and S-*tert*-butylation slightly increased the MIC of NFAPimpγ and NFAPimpγGZ from 12.5 to 50 μg mL^–1^ and from 12.5 to 25 μg mL^–1^ against *C. albicans*, respectively. It was partially true for *A. fumigatus*, where MICs of cysteine–serine substituted variants of NFAPimpγGZ increased from 25 to 50 μg mL^–1^. However, S-*tert*-butylation did not change the efficacy of this peptide (MIC: 25 μg mL^–1^). Interestingly, S-*tert*-butylation made the NFAPimpγ peptide antifungal active against this fungus. These data are summarized in Table 2.

**Table 2:** Antifungal Efficacy of NFAP γ-Core Peptide Derivatives Prompt after the Synthesis and Lyophilization Stored at −20 °C and after Storing at 4 °C in an Aqueous Solution for 7 days

peptide	MIC (μg mL^–1^)^a^			
	*A. fumigatus* CBS 101355		*C. albicans* SC5314	
	–20 °C sample	4 °C sample	–20 °C sample	4 °C sample
NFAPimpγ	>200	>200	12.5	25
NFAPimpγ^C6S,C13S^	>200	>200	50	50
NFAPimpγ^C13S^	>200	>200	50	50
NFAPimpγ^S-*t*Bu^	25	25	50	50
NFAPimpγGZ	25	25	12.5	12.5
NFAPimpγGZ^C6S,C13S^	50	50	25	25
NFAPimpγGZ^C13S^	50	50	25	25
NFAPimpγGZ^S-*t*Bu^	25	25	25	25

a MIC: minimum inhibitory concentration. MIC was defined as the lowest peptide concentration at which growth was ≤5% compared with the untreated control.

The antifungal activities of the designed synthetic NFAP γ-core peptide derivatives against yeasts and *A. fumigatus* have not been previously tested. Our results coincide with previous observations that the peptide spanning the native NFAP γ-core motif is inactive, and the positive net charge can render the peptide antifungal active.^16^ However, some of our results contradict the previous observations, that the primary structure and hydrophobicity do not support the antifungal efficacy of NFAPimpγ.^16,19^ For example, most NFAPimpγ peptides did not inhibit the growth of *A. fumigatus* but all NFAPimpγGZ did. However, NFAPimpγ and NFAPimpγGZ peptides have the same positive net charge (+5.8 or +5.9), but NFAPimpγGZ and all its variants have increased hydrophobicity and different primary structures (Table 1). S-*tert*-Butylation increases the GRAVY, which may make the NFAPimpγ active against *A. fumigatus* and help maintain the antifungal efficacy of NFAPimpγGZ against this fungus. These findings with the previous observation by Tóth *et al.* (2020)^16^ indicate that hydrophobicity is not as crucial a feature for AFPs as for peptides with antibacterial activity, where it is required for bacterial membrane permeabilization;^21^ however, it can be important depending on the fungal species.

Cysteine–serine substituted NFAP γ-core peptide variants indicated that disulfide bridge formation between the cysteine residues is not required for antifungal activity but can affect the efficacy, as previously observed in several antimicrobial peptides.^22−26^ In our study, this substitution resulted in decreased antifungal efficacy, as indicated by increased MICs. In the literature, examples can be found when cysteine–serine substitution retained,^22,23^ increased,^26^ or decreased the efficacy of an antimicrobial peptide.^22−25^ The nature of this effect highly depended on the tested microorganism. Generally, cysteine–serine substitution does not change or increase the antimicrobial efficacy against Gram-positive bacteria;^22−25^ however, it decreases it against Gram-negative ones and yeasts.^22,23^ According to Imamura *et al.* (2008),^23^ introducing the *S*-*tert*-butyl group into cysteine residues enhanced the activity of an antibacterial peptide, thanatin against the Gram-positive bacterium, *Micrococcus luteus*. Here we had a similar observation to the antifungal peptide NFAPimpγ against *A. fumigatus* (Table 2).

### 2.4 *Candida albicans* Cell-Killing Efficacy

Compared with MIC determination, monitoring the growth kinetics of high-density cell cultures in the presence of various peptide concentrations allows for detailed insight into the cell-killing efficacy over time and how cysteine modifications influence it. Therefore, the growth of high-density *C. albicans* cell culture (OD_600_ = 0.2) was monitored in the presence of 0.5× MIC, 1× MIC, and 2× MIC of the NFAP γ-core peptides.

**Figure 3:**
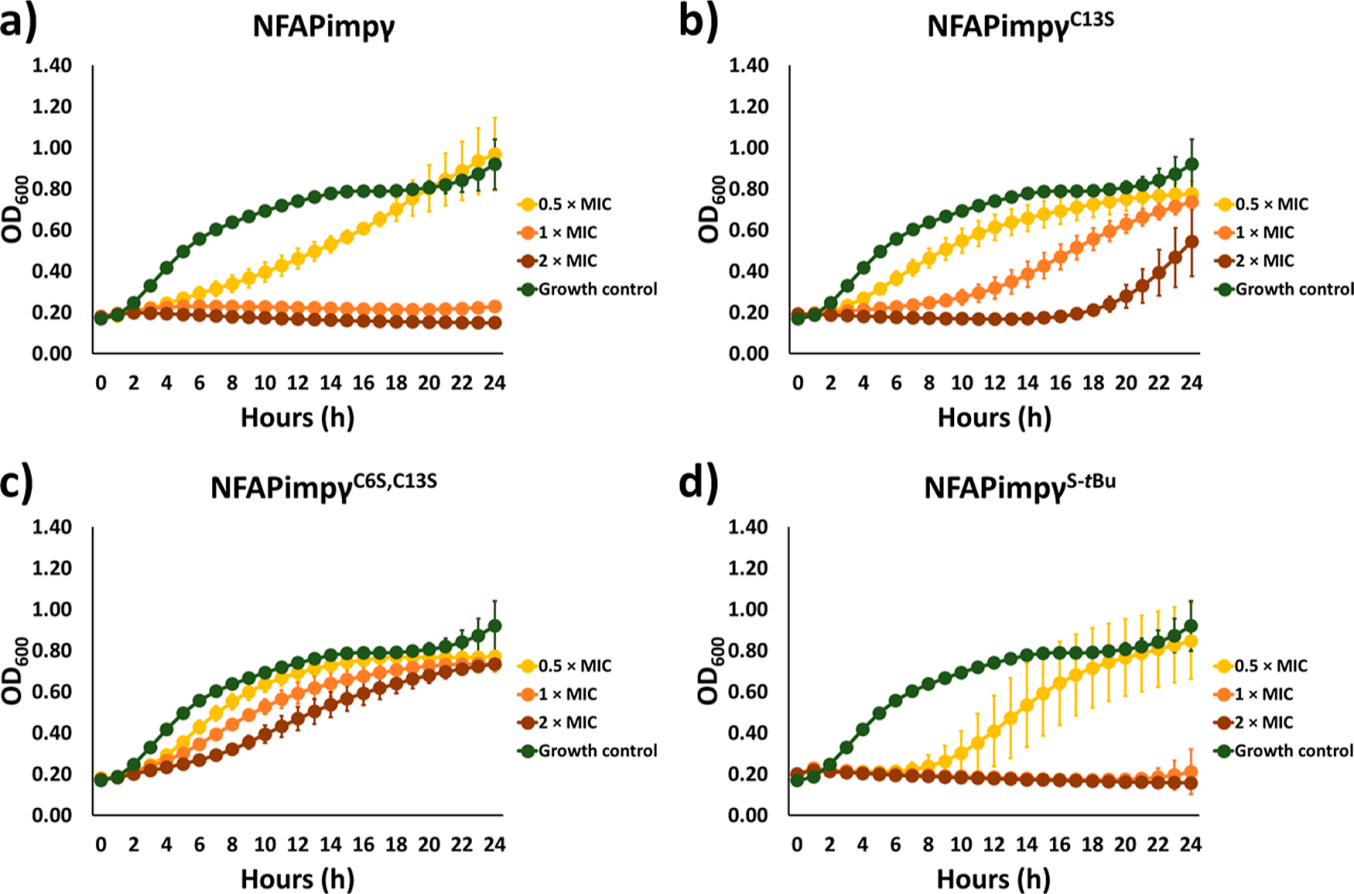
Time-kill curves of various NFAPimpγ peptides (a–d) against *C. albicans* SC5314.

According to the growth curves (Figure 3a,c), cysteine–serine substitutions impaired the antifungal efficacy of NFAPimpγ against *C. albicans*. The 1 and 2× MIC of NFAPimpγ^C13S^ and NFAPimpγ^C6S,C13S^ could not fully inhibit the growth (Figure 3b,c), as previously observed for the unmodified variant, NFAPimpγ (Figure 3a). The single C13S substitution did not decrease the antifungal efficacy as dramatically as the double C6S, C13S substitution (Figure 3b,c). This was indicated as the *Candida* cells started to grow intensively in the presence of 0.5× MIC and 1× MIC of NFAPimpγ^C13S^ compared with NFAPimpγ^C6S,C13S^ (Figure 3b,c). S-*tert*-Butylation made this peptide a little more effective (Figure 3d). The 0.5× MIC of NFAPimpγ^S-*t*Bu^ extended the time when the *C. albicans* culture started to multiply intensively compared with the unmodified NFAPimpγ variant (Figure 3a,d).

**Figure 4:**
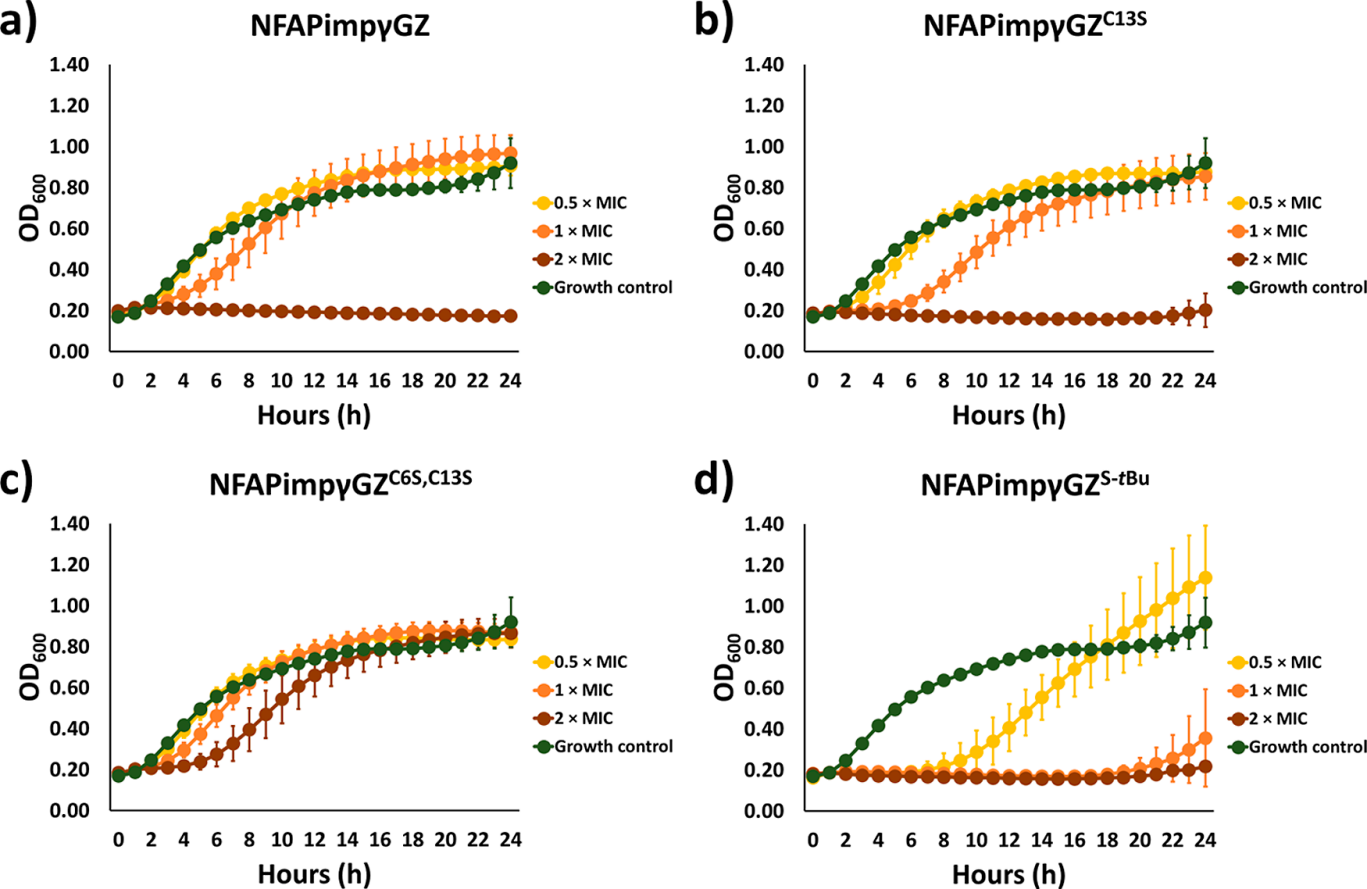
Time-kill curves of various NFAPimpγGZ peptides (a–d) against *C. albicans* SC5314.

For NFAPimpγGZ, the C13S substitution did not influence the antifungal efficacy against *C. albicans* (Figure 4a,b). However, the C6S, C13S substitution greatly decreased it (Figure 4a,c). The 2× MIC of NFAPimpγGZ^C6S,C13S^ could not inhibit the growth of *C. albicans* at that concentration (Figure 4c), where total or high growth inhibitions were reached with the unmodified (NFAPimpγGZ) and C13S (NFAPimpγGZ^C13S^) variants (Figure 4a,b). Conversely, NFAPimpγGZ^S-*t*Bu^ was more effective than unmodified NFAPimpγGZ (Figure 4a,d). The 1× MIC of NFAPimpγGZ^S-*t*Bu^ inhibited the growth, whereas this concentration of NFAPimpγGZ allowed the multiplication of *C. albicans* cells within 24 h (Figure 4a,d).

Growing abilities of the culture drops from the above-mentioned antifungal efficacy experiments indicated that none of the NFAP γ-core peptides killed all *C. albicans* cells in the wells; however, treatments with 2× MIC NFAPimpγ, 1–2× MIC NFAPimpγ^S-*t*Bu^, 2× MIC NFAPimpγGZ, and 2× MIC NFAPimpγGZ^S-*t*Bu^ could highly reduce the proportion of cells able to multiply (Figure S4).

The outcome of antimicrobial susceptibility tests is highly dependent on the number of applied cells. The reason for the lack of full growth inhibition in the cell-killing efficacy experiment compared with the MIC determination is the increase in the number of cells with one magnitude. However, all of the above results supported the antifungal susceptibility test (MIC) results with C13S and C6S, C13S peptide variants. They indicated that the presence of cysteine residue(s) is required for the high anti-*Candida* efficacy of NFAP γ-core peptide derivatives. As previously stated, cysteine–serine substitution(s) can impair, enhance, or have no effect on the efficacy of an antimicrobial peptide.^22−26^ Based on the previous observations, the nature of this effect may depend on the primary structure of the peptide and the microorganism tested. According to the growth curves, S-*tert*-butylation made the NFAP γ-core peptide derivatives more antifungal active against *C. albicans* (Figures 3a,d and 4a,d), similar to an S-*tert*-butylated antimicrobial peptide, which was more active than the unmodified variant.^23^ Somehow, this observation contradicts the MIC results from the susceptibility tests (Table 2). However, it is worth noting that the growth curves were monitored for 24 h and the susceptibility was determined after 48 h of incubation. Ascendant growth curves in the presence of NFAPimpγ^S-*t*Bu^ and NFAPimpγGZ^S-*t*Bu^ and higher cell density in the presence of 0.5× MIC of NFAPimpγGZ^S-*t*Bu^ at the 24th hour than that of the growth control were observed (Figures 3a,d and 4a,d). These indicated that *Candida* cells may overcome the higher growth inhibitory effect of the S-*tert*-butylated peptide variants over time.

### 2.5 Hemolysis and Toxicity

The clinical application of antimicrobial peptides is limited by their potential hemolytic activity, toxicity, immunogenicity, and other side effects.^27^ In our previous study, we demonstrated that NFAPimpγ and NFAPimpγGZ did not cause hemolysis and showed that they are not toxic to various human cell lines (such as monocytes, colonic epithelial cells, and keratinocytes), except for NFAPimpγGZ, which impaired the viability of keratinocytes at higher concentrations.^16^ Herein, we investigated how cysteine–serine substitution(s) and S-*tert*-butylation of these peptides influence hemolytic activity and *in vivo* toxicity.

The potential human cell membrane disruption ability of the NFAP γ-core peptide derivatives was investigated on sheep blood agar plates. The hemolysis test confirmed our previous results that neither the NFAPimpγ nor NFAPimpγGZ peptides cause hemolysis^16^ and provided new information that cysteine–serine substitutions and S-*tert*-butylation did not make the peptides hemolytically active (Figure S5). These results suggest that the elevated hydrophobicity of NFAPimpγGZ peptides and S-*tert*-butylation cannot lead to mammalian cell membrane disruption contrary to antimicrobial peptides with antibacterial activity where it can happen.^21^

A well-established and described acute toxicity test in *Galleria mellonella* (greater wax moth) larvae was conducted to reveal the potential *in vivo* harmful effects of NFAP γ-core peptide derivatives in animals.^28^ According to the survival analysis of the larvae injected with peptides, none of the tested peptides proved to be toxic (Figure S6).

All of the above results show that cysteine–serine substitution(s) and S-*tert*-butylation apparently do not change the fungal selectivity of NFAP γ-core peptide derivatives. However, the outcome of the pilot *in vitro* and *in vivo* toxicity tests with antimicrobial peptides can depend on the toxicity model applied.^29^

## 3 Conclusions

In this paper, we demonstrated that the substitutions of all cysteines with serines and S-*tert*-butylation of cysteine residues help maintain the structural integrity of AFPs designed on the evolutionarily conserved γ-core region of an antifungal protein (NFAP) from mold *N.* (*A.*) *fischeri*. These modifications inhibited the cyclization and dimerization of peptides by disulfide bond formation between cysteine residues. However, we observed that the structural integrity did not influence the antifungal efficacy of most of these peptides. The NFAP γ-core peptide derivatives proved to be antifungal active against the tested yeast (*C. albicans*); however, their increased hydrophobicity made them antifungal active against the tested mold (*Aspergillus fumigatus*). Our results revealed that cysteine–serine substitution(s) decreased the antifungal efficacy against *C. albicans*. Furthermore, S-*tert*-butylation could decrease the antifungal efficacy against this yeast for a long time. However, these peptide variants could be more effective for a short time and have extended the antifungal spectrum to *A. fumigatus* or helped maintain the efficacy against this mold. None of the generated peptides proved to be hemolytically active and harmful in the *G. mellonella* toxicity model, indicating the potential safe applicability. Based on the above-mentioned results, we suggest synthesizing AFPs with *S*-*tert*-butyl-protected cysteine residues to maintain structural integrity, widen the antifungal spectrum, and make them more effective in the short term.

## 4 Methods

### 4.1 *In Silico* Analyses

Physicochemical properties of the designed NFAP γ-core peptide derivatives were predicted using the ExPASy ProtParam tool (molecular weight, *M*_w_; pI, and GRAVY)^30^ and Protein Calculator v3.4 server (total net charge at pH = 7.0) (The Scripps Research Institute; http://protcalc.sourceforge.net).

### 4.2 Peptide Synthesis

Peptides were synthesized by microwave-assisted, stepwise solid-phase peptide synthesis using Fmoc/S-*t*Bu chemistry and a Liberty Blue peptide synthesizer (CEM Corporation, Matthews, NC, USA). Syntheses were performed on TentaGel S RAM resin (substitution level: 0.2 mmol g^–1^) applying ethyl 2-cyano-2-(hydroxyimino)acetate (Oxyma)/diisopropylcarbodiimide (DIC) coupling. Peptides were cleaved off the resin with a trifluoroacetic acid (TFA)/water/dithiothreitol (DTT) (95%:5%:3% v/v:v/v:m/v) cocktail in 3 h. TFA was removed by evaporation, and the peptides were precipitated with ice-cold diethyl ether, dissolved in a 10% (v/v %) acetic acid solution, and then lyophilized. The lyophilized peptides were stored at −20 °C until further analyses.

### 4.3 Peptide Sample Preparation

The lyophilized and stored peptides were dissolved in sterile ddH_2_O (5 mg mL^–1^). Then, half of the volume of the peptide stock was tested for antifungal activity and stored at −20 °C until RP-HPLC and ECD analyses. This peptide sample is termed the −20 °C sample. The other half volume of the stock was stored at 4 °C for 7 days, tested for antifungal activity, and stored at −20 °C until RP-HPLC and ECD analyses. This peptide sample is referred to as the 4 °C sample. A schematic representation of the sample preparation is shown in Figure S7.

### 4.4 Reversed-Phase High-Performance Liquid Chromatography Analysis

Crude peptides were purified by semipreparative RP-HPLC using a solvent system of (A) 0.1% TFA and (B) 80% acetonitrile, 0.1% TFA, and a linear gradient from 0 to 30% (B) in 60 min. Purification was performed on a Phenomenex Jupiter Proteo 90 Å column (250 × 10 mm) using a Shimadzu HPLC apparatus (Berlin, Germany). Absorbance was detected at 220 nm. Purity was evaluated by analytical RP-HPLC using a Phenomenex Luna 10 μ C18 100 A column and an Agilent 1100 HPLC instrument (Palo Alto, CA, USA).

### 4.5 ECD Analysis

The secondary structure of the NFAP γ-core peptide and its derivatives was examined by ECD spectroscopy. ECD spectra were acquired in the wavelength range of 195–260 nm using a Jasco-J815 spectropolarimeter (JASCO, Tokyo, Japan). Peptide samples, prepared and stored as described above, were diluted to 0.1 mg mL^–1^ concentration with bidistilled H_2_O and transferred into a 0.1 cm path-length quartz cuvette for ECD measurements. Samples were kept at a constant 25 °C temperature during measurements using a Peltier thermoelectric controller (TE Technology, Traverse City, MI, USA). The presented spectra are accumulations of ten scans for each sample from which the corresponding solvent spectra, recorded under similar conditions, were subtracted. The ellipticity data are presented in molar ellipticity units. Spectral contributions emerging from specific canonical secondary structural states were estimated using the DichroWeb server and the CDSSTR method.^31,32^

### 4.6 Antifungal Susceptibility Test

MICs of NFAP γ-core peptide derivatives were determined against *C. albicans* SC5314 and *A. fumigatus* CBS 101355. These fungi were maintained on yeast peptone dextrose agar (YPD: 10 g L^–1^ yeast extract, 20 g L^–1^ peptone, 20 g L^–1^d-glucose, 15 g L^–1^ agar) and malt extract agar (MEA, Sigma-Aldrich) slants at 4 °C, respectively. Susceptibility tests were performed in low cationic medium (LCM: 5 g L^–1^d-glucose, 0.25 g L^–1^ yeast extract, 0.125 g L^–1^ peptone) according to Tóth *et al.* (2018)^16^ and Tóth *et al.* (2020)^19^ for yeasts and molds, respectively, with slight modifications. Briefly, 100 μL NFAP γ-core peptide (0.39–400 μg mL^–1^ in two-fold serial dilutions in LCM) was mixed with 100 μL of 2 × 10^5^ mL^–1^ mid-log phase *C. albicans* cells or freshly harvested *A. fumigatus* conidia in LCM in a flat-bottom 96-well microtiter plate (TC Plate 96 Well, Suspension, F; Sarstedt, Nümbrecht, Germany). A mixture of 100 μL of medium (LCM) without NFAP γ-core peptide derivative and 100 μL of cell or conidium suspension served as the untreated growth control, whereas 200 μL of LCM was used for background calibration. The plates were incubated statically for 48 h (*C. albicans*) or 72 h (*A. fumigatus*) at 30 °C. The absorbance (OD_620_) of each well was measured after shaking for 5 s using a microtiter plate reader (SPECTROstar Nano; BMG Labtech, Ortenberg, Germany). The absorbance of the untreated control was taken as 100% growth for the MIC calculation. MIC was defined as the lowest peptide concentration that reduced fungal growth by ≤5% compared with the untreated control. Susceptibility tests were performed in two technical replicates and were repeated at least two times.

### 4.7 Antifungal Efficacy Tests

To examine the yeast cell-killing efficacy of the generated NFAP γ-core peptide derivatives, the growth ability of *C. albicans* SC5314 was monitored in the presence of 0.5× MIC, 1× MIC, and 2× MIC of the peptides that proved to be effective in the antifungal susceptibility tests. The experiment was conducted in a flat-bottom 96-well microtiter plate (TC Plate 96 Well, Suspension, F; Sarstedt, Nümbrecht, Germany): 100 μL of OD_600_ = 0.2 mid-log phase cells prepared in LCM were mixed with 100 μL of 1× MIC, 2× MIC, or 4× MIC of the peptide diluted in LCM. The plates were incubated statically at 30 °C for 24 h. The absorbance (OD_600_) of each well was then measured after shaking for 5 s every hour using a microtiter plate reader (BioTek Synergy HTX Multi-Mode Microplate Reader; Agilent Technologies, Santa Clara, CA, USA). Untreated cells (100 μL LCM mixed with 100 μL OD_600_ = 0.2 mid-log phase cells prepared in LCM) served as growth controls. To examine the viability of the treated *C. albicans* cells, 5–5 μL cultures from each well were dropped onto the YPD agar plate after the last OD_600_ measurement and allowed to dry before incubation at 30 °C for 24 h. The plates were photographed (Versa Doc Imaging System 4000 MP; Bio-Rad, Hercules, CA, USA). These experiments were repeated two times, involving three technical replicates.

### 4.8 Hemolysis Assay

The hemolytic activity of NFAP γ-core peptide derivatives was tested on Columbia blood (5% (v/v %) sheep blood) agar plates (VWR; Radnor, PA, USA). Sterile filter paper disks (*Ø* 6 mm) with 10 μL drops of NFAP γ-core peptide derivatives (1 mg mL^–1^) were placed on the agar plates. Sterile ddH_2_O and 20% (v/v) Triton X-100 were used as negative and positive hemolysis controls, respectively. The plates were incubated for 24 h at 37 °C and examined for the presence of clear zones around the filter disks. The experiment was performed in three technical replicates.

### 4.9 *G. mellonella* Toxicity Assay

To check the potential *in vivo* toxic effect of the generated NFAP γ-core peptide derivatives, a *G. mellonella* toxicity assay was applied. This animal model is not subject to ethical considerations.^28^ Twenty microliter peptides solution (200 μg mL^–1^) prepared in insect physiological saline (IPS: 50 mM NaCl, 5 mM KCl, 10 mM EDTA, and 30 mM sodium citrate in 0.1 M Tris-HCl; pH 6.9) were injected intrahemocoelicly (29-gauge insulin needles; BD Micro-Fine, Franklin Lakes, NJ, USA) through the last right pro-leg of 20 larvae. Then, the larvae were incubated at 37 °C, and the survival was monitored every 24 h for 6 days. IPS-treated larvae served as nontoxic, 20% (v/v) Triton X-100-treated ones as positive toxicity, and larvae without any interventions as untreated controls. The toxicity assay was repeated 2 times.

### 4.10 Statistical Analysis

Statistical analyses were performed using GraphPad Prism 7.00 software (GraphPad Software, Boston, MA, USA). Log-rank (Mantel–Cox) and Gehan–Breslow–Wilcoxon tests (software) were used to compare the survival curves in the *G. mellonella* larva toxicity assay. The toxic effect of the peptide was considered significant if *p* ≤ 0.05 in both tests.

## Supporting Information Available

Estimation of ECD spectral contributions emerging from canonical secondary structural elements using the secondary structure from circular dichroism spectra (CDSSTR) method; RP-HPLC elution profiles of −20 and 4 °C samples of NFAPγ peptide and its C13S, C13S, C16S, and S-*tert*-butylated variants; RP-HPLC elution profiles of −20 and 4 °C samples of NFAPimpγ peptide and its C13S, C13S, C16S, S-*tert*-butylated variants; RP-HPLC elution profiles of −20 and 4 °C samples of NFAPimpγGZ peptide and its C13S, C13S, C16S, and S-*tert*-butylated variants; growth abilities of *C. albicans* SC5314 mid-log phase cell cultures on YPD agar plate after treatment with different concentrations of NFAP γ-core peptides; hemolytic activity of NFAP γ-core peptide derivatives in aqueous solution on Columbia blood agar plates; and survival of *G. mellonella* larvae after injection with NFAP γ-core peptide derivatives (PDF)
