# Supplementary material for: Rational Design of Antifungal Peptides Based on the γ-Core Motif of a Neosartorya (Aspergillus) fischeri Antifungal Protein to Improve Structural Integrity, Efficacy, and Spectrum
Source: ACS Omega. 2024 Jan 31;9(6):7206–14. doi: 10.1021/acsomega.3c09377 (PMC10870298; doi:10.1021/acsomega.3c09377)
Supplement: Supplementary file 1 — ao3c09377_si_001.pdf [file ao3c09377_si_001.pdf]

Supporting Information

Rational design of antifungal peptides based on the γ-core motif of a *Neosartorya* (*Aspergillus*) *fischeri* antifungal protein to improve structural integrity, efficacy, and spectrum

Györgyi Váradi[1], Gábor Bende[2,3], Attila Borics[4], Kinga Dán[2,3], Gábor Rákhely[2,5], Gábor K. Tóth[1,6], László Galgóczy[2,4]

[1]Department of Medical Chemistry, University of Szeged, Szeged 6720, Hungary
[2]Department of Biotechnology, University of Szeged, Szeged 6726, Hungary
[3]Doctoral School of Biology, University of Szeged, Szeged 6720, Hungary
[4]Institute of Biochemistry, HUN-REN Biological Research Centre, Szeged 6726, Hungary
[5]Institute of Biophysics, HUN-REN Biological Research Centre, Szeged 6726, Hungary
[6]MTA-SZTE Biomimetic Systems Research Group, University of Szeged, Szeged 6720, Hungary

Table S1. Estimation of ECD spectral contributions emerging from canonical secondary structural elements using the Secondary Structure from Circular Dichroism Spectra (CDSSTR) method.

Peptide	Sample	Helix1	Helix2	Strand1	Strand2	Turns	Unordered	Total
**NFAPγ**	4ºC	-0.01	0.12	0.16	0.09	0.25	0.38	0.99
	−20ºC	-0.00	0.12	0.17	0.09	0.27	0.34	0.99
**NFAPγ^{C13S}**	4ºC	-0.02	0.11	0.16	0.10	0.24	0.40	0.99
	−20ºC	-0.02	0.12	0.14	0.12	0.29	0.32	0.97
**NFAPγ^{C6S,C13S}**	4ºC	-0.02	0.12	0.19	0.10	0.22	0.37	0.98
	−20ºC	-0.01	0.10	0.18	0.10	0.23	0.39	0.99
**NFAPγ$^{S\text{-}tBu}$**	4ºC	0.00	0.13	0.16	0.08	0.24	0.37	0.98
	−20ºC	-0.01	0.12	0.17	0.09	0.24	0.38	0.99
**NFAPimpγ**	4ºC	-0.01	0.08	0.19	0.11	0.24	0.37	0.98
	−20ºC	-0.01	0.10	0.17	0.10	0.25	0.38	0.99
**NFAPimpγ^{C13S}**	4ºC	-0.03	0.08	0.17	0.10	0.27	0.38	0.97
	−20ºC	-0.02	0.12	0.13	0.11	0.30	0.34	0.98
**NFAPimpγ^{C6S,C13S}**	4ºC	-0.01	0.08	0.19	0.11	0.24	0.37	0.98
	−20ºC	-0.03	0.10	0.20	0.10	0.22	0.39	0.98
**NFAPimpγ$^{S\text{-}tBu}$**	4ºC	-0.01	0.11	0.17	0.09	0.23	0.38	0.97
	−20ºC	-0.01	0.10	0.16	0.10	0.26	0.38	0.99
**NFAPimpγGZ**	4ºC	0.06	0.11	0.14	0.11	0.28	0.31	1.01
	−20ºC	-0.01	0.14	0.15	0.09	0.25	0.37	0.99
**NFAPimpγGZ^{C13S}**	4ºC	0.01	0.13	0.12	0.08	0.29	0.37	1.00
	−20ºC	-0.01	0.13	0.16	0.10	0.26	0.35	0.99
**NFAPimpγGZ^{C6S,C13S}**	4ºC	-0.02	0.09	0.17	0.11	0.22	0.41	0.98
	−20ºC	-0.02	0.12	0.15	0.09	0.22	0.43	0.99
**NFAPimpγGZ$^{S\text{-}tBu}$**	4ºC	0.04	0.12	0.15	0.09	0.26	0.34	1.00
	−20ºC	0.00	0.14	0.16	0.08	0.23	0.39	1.00

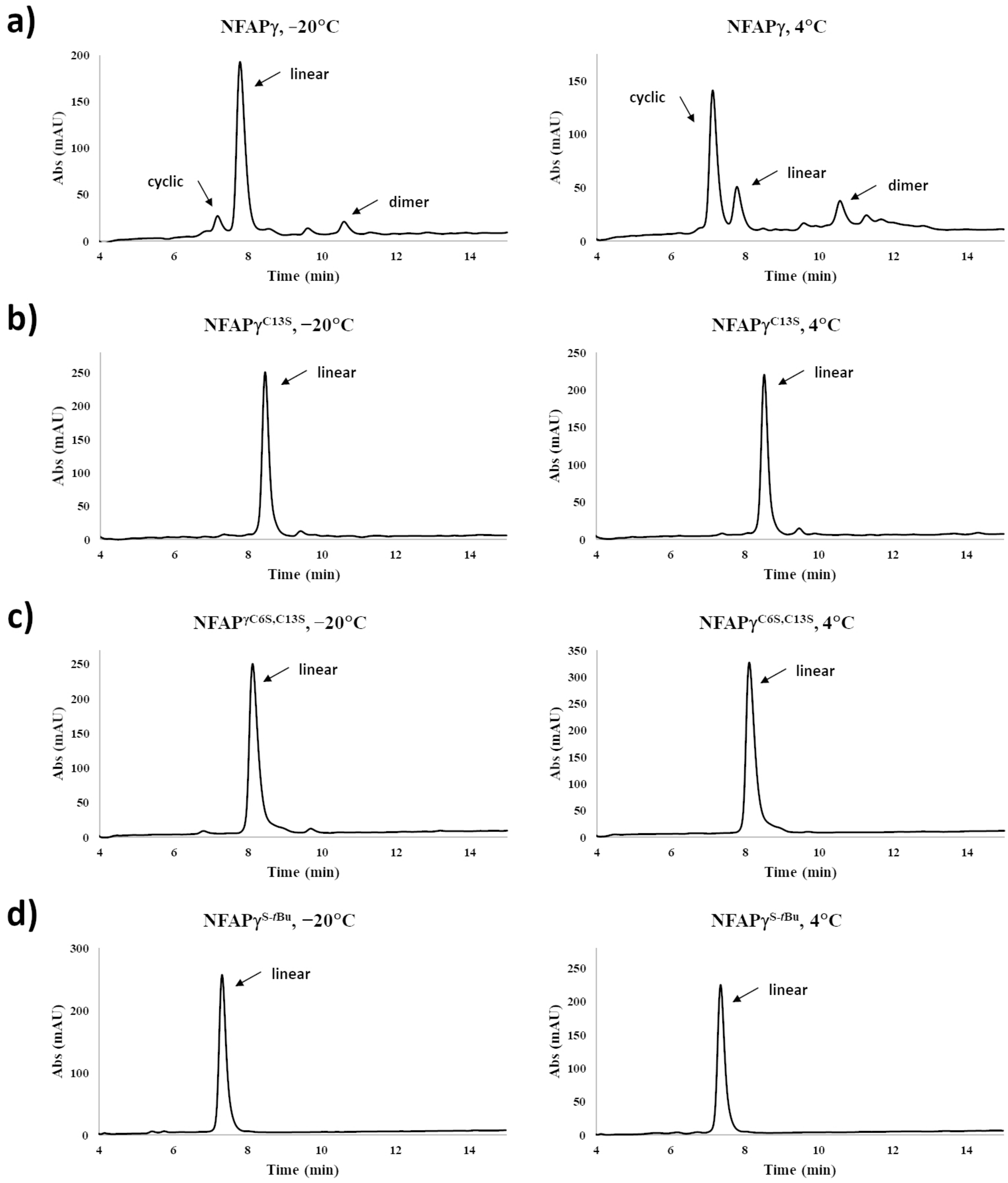

Figure S1. RP-HPLC elution profiles of −20°C and 4°C samples of NFAPγ peptide (**a**) and its C13S (**b**), C13S,C16S (**c**), and S-*tert*-butylated (S-*t*Bu) (**d**) variants. The following linear gradients of eluent B were used in 15 min: 19-34% (**a**), 17-32% (**b**), 16-31% (**c**), and 35-50% (**d**).

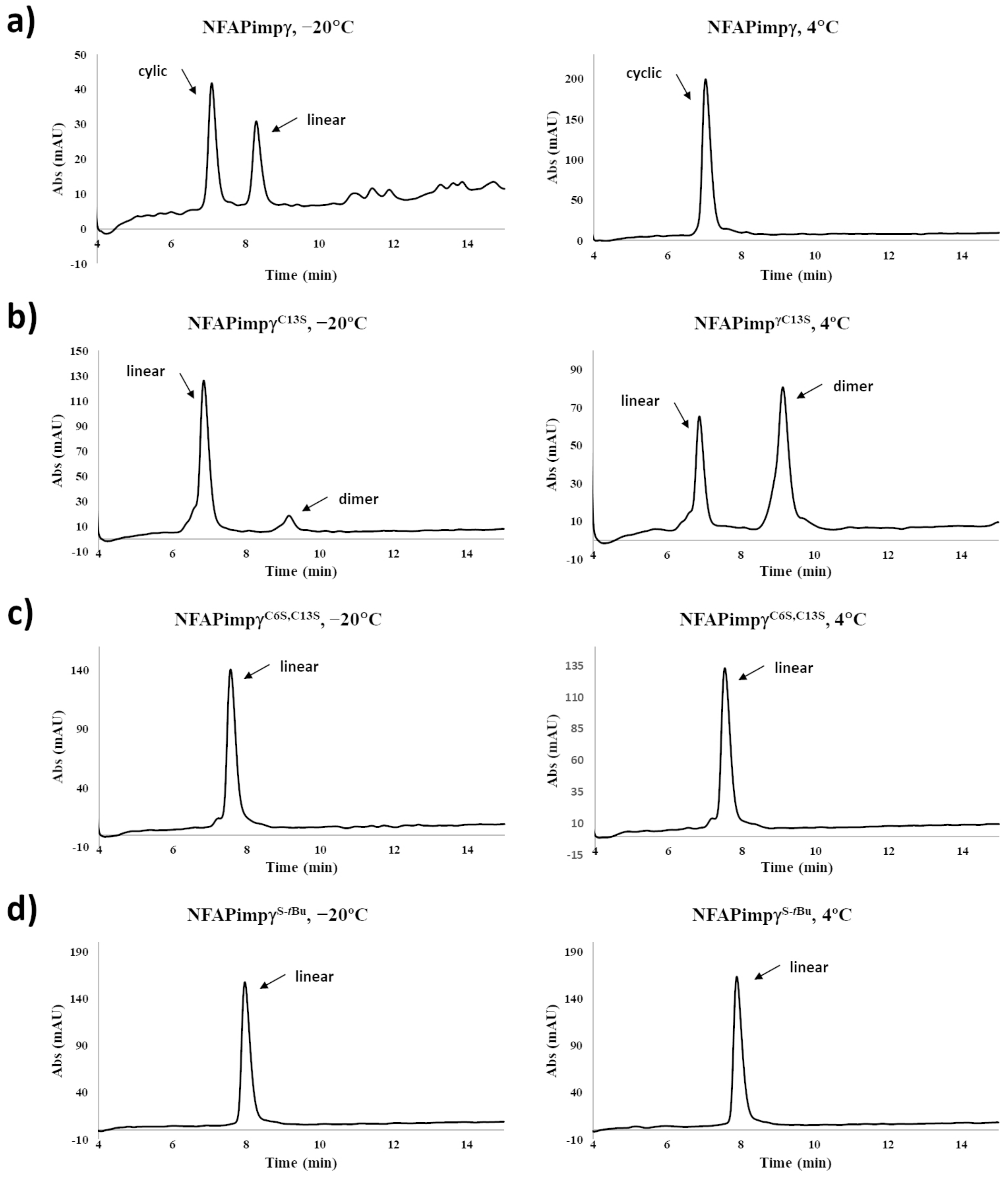

Figure S2. RP-HPLC elution profiles of −20°C and 4°C samples of NFAPimpγ peptide (**a**) and its C13S (**b**), C13S,C16S (**c**), S-*tert*-butylated (S-*t*Bu) (**d**) variants. The following linear gradients of eluent B were used in 15 min: 10-25% (**a**), 11-26% (**b**), 9-24% (**c**), and 23-38% (**d**).

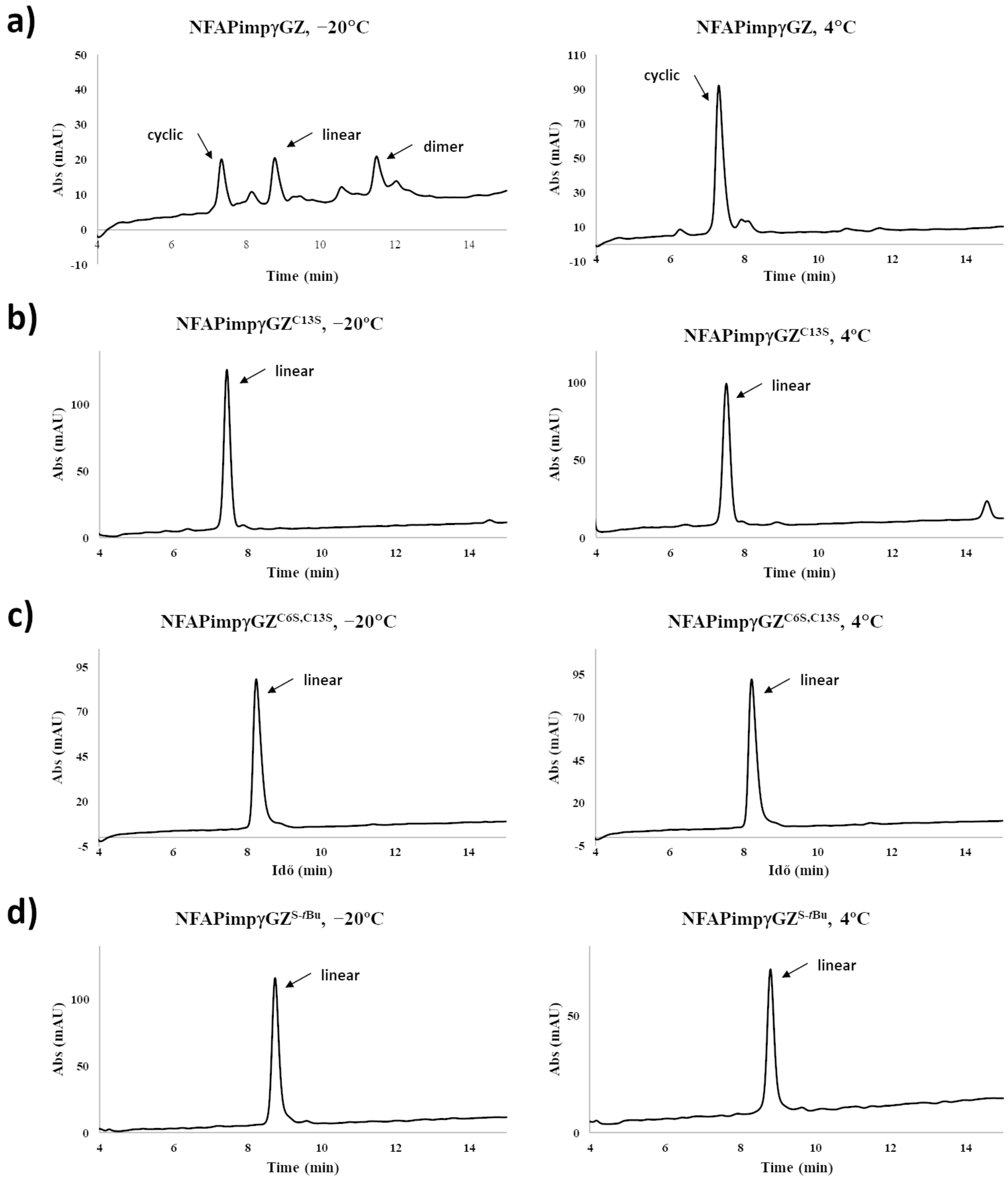

Figure S3. RP-HPLC elution profiles of −20°C and 4°C samples of NFAPimpγGZ peptide (**a**) and its C13S (**b**), C13S,C16S (**c**), and S-*tert*-butylated (S-*t*Bu) (**d**) variants. The following linear gradients of eluent B were used in 15 min: 22-37% (**a**), 23-38% (**b**), 21-36% (**c**), and 24-39% (**d**).

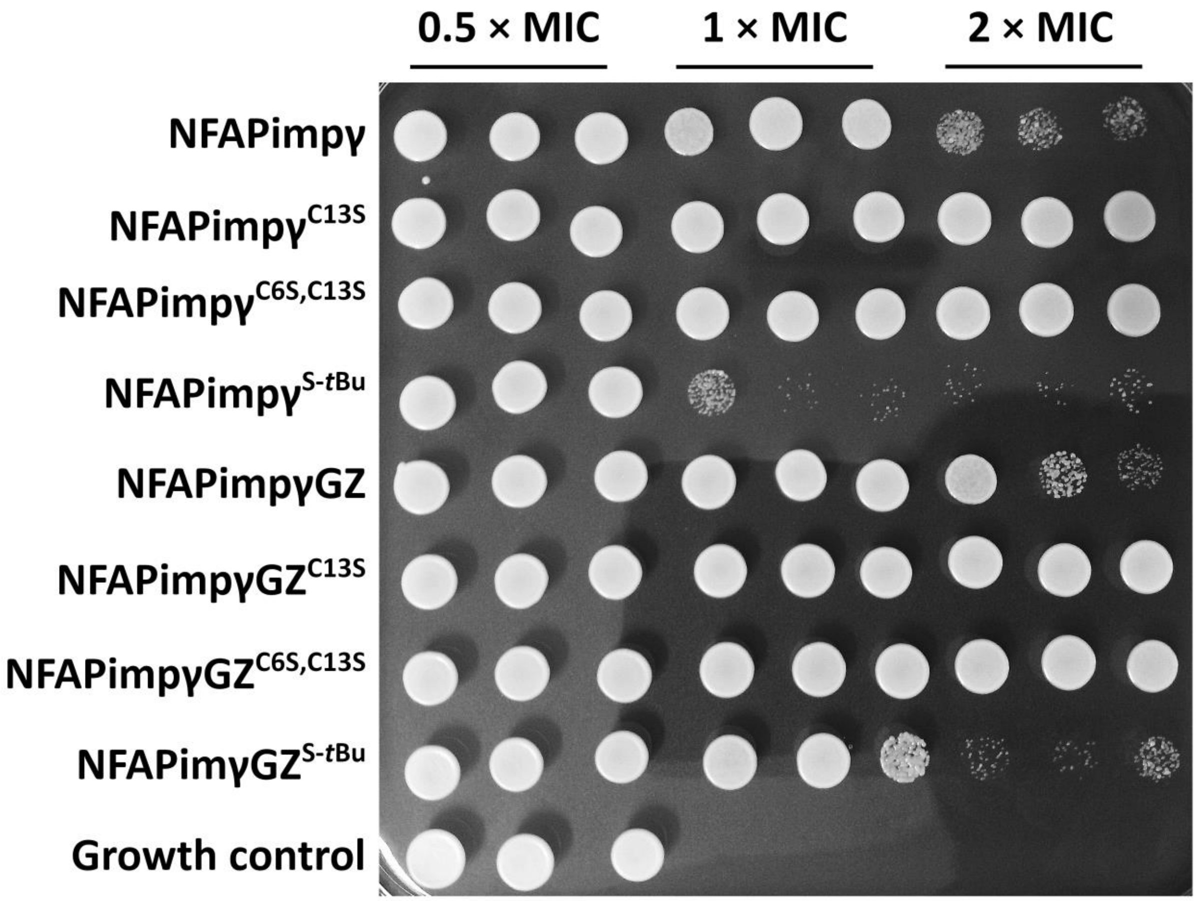

Figure S4. Growth abilities of *Candida albicans* SC5314 mid-log phase cell cultures (OD_{600} = 0.2) on YPD agar plate after treatment with different concentrations of NFAP γ-core peptides at 30°C for 24 hours. MIC: minimum inhibitory concentration (Table 2).

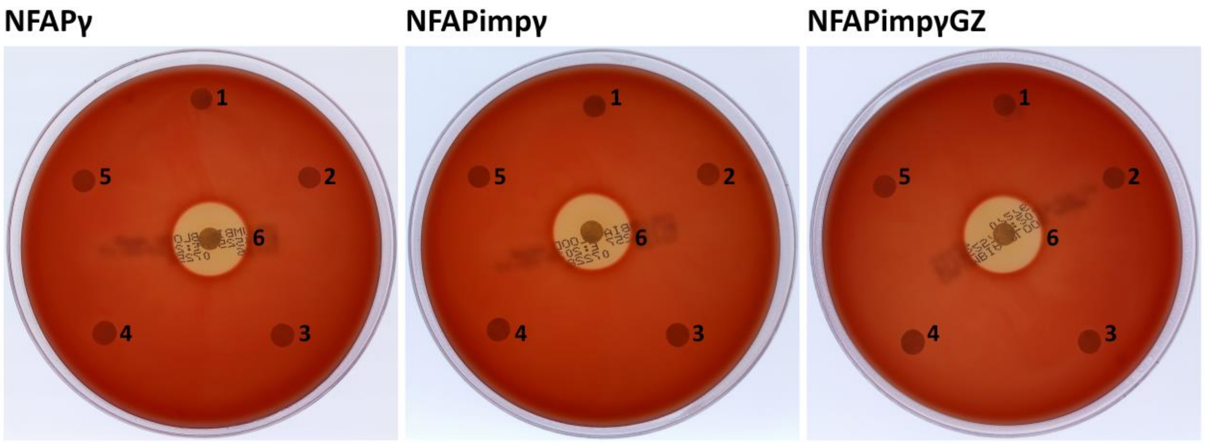

Figure S5. Hemolytic activity of 10 μg NFAP γ-core peptide derivatives in aqueous solution on Columbia blood agar plates after incubation for 24 h at 37°C. Triton X-100 [20% (v/v)] and ddH_2O were used as the positive and negative lysis controls, respectively. 1: ddH_2O; 2: unmodified peptide, 3: C13S variant, 4: C6S,C13S variant, 5: S-*tert*-butylated variant, 6: Triton X-100 [20% (v/v)].

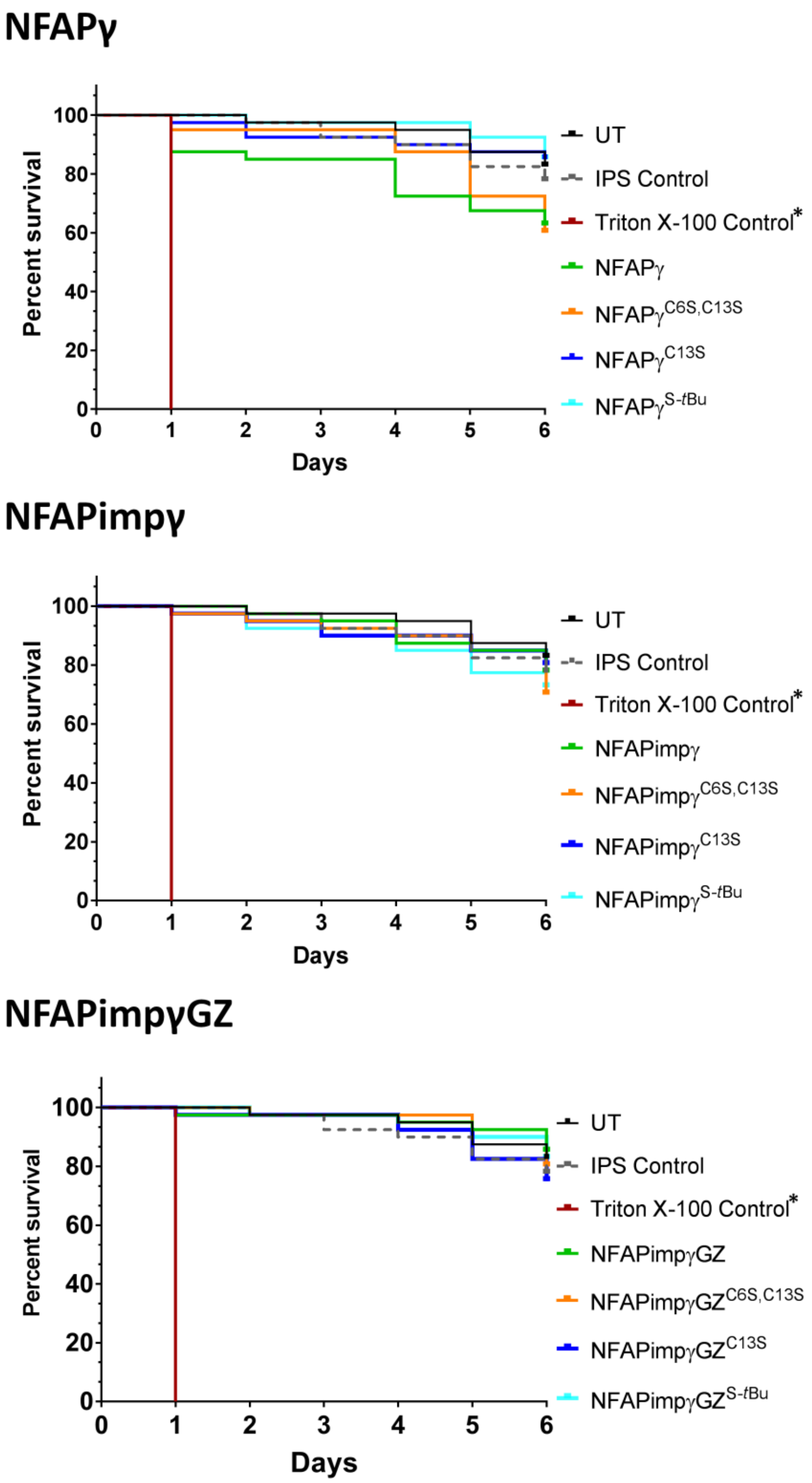

Figure S6. Survival of *Galleria mellonella* larvae after injection with NFAP γ-core peptide derivatives (20 µL from 200 µg mL^{-1} solution) in comparison with the untreated control. UT: untreated control, IPS: insect physiological saline-treated control. *: $p \leq 0.05$ from both Log-rank (Mantel-Cox) and Gehan-Breslow-Wilcoxon tests.

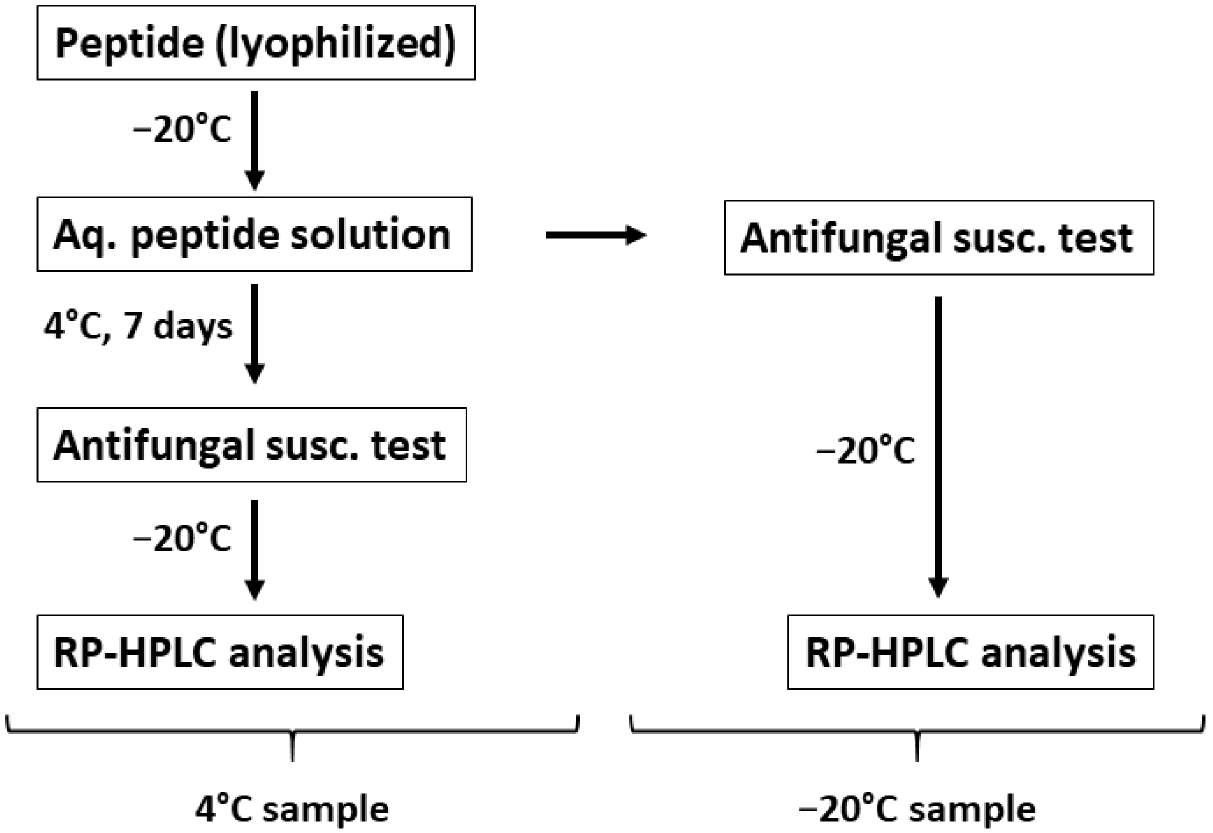

Figures S7. Schematic representation of the peptide sample preparation for antifungal susceptibility testing, RP-HPLC, and ECD analyses.
